# Potential effect of key soil bacterial taxa on the increase of rice yield under milk vetch rotation

**DOI:** 10.3389/fmicb.2023.1150505

**Published:** 2023-05-22

**Authors:** Mingming Xia, Xinling Ma, Jia Liu, Meng Wu, Zhongpei Li, Ming Liu

**Affiliations:** ^1^State Key Laboratory of Soil and Sustainable Agriculture, Institute of Soil Science, Chinese Academy of Sciences, Nanjing, China; ^2^University of Chinese Academy of Sciences, Beijing, China; ^3^National Engineering and Technology Research Center for Red Soil Improvement, Soil and Fertilizer & Resources and Environment Institute, Jiangxi Academy of Agricultural Sciences, Nanchang, China; ^4^Ecological Experimental Station of Red Soil Academia Sinica, Nanjing, China

**Keywords:** milk vetch rotation, functional genes, phosphorus metabolism, crop yield, latent phosphate-solubilizing microorganisms

## Abstract

Legume crop rotation is often adopted in rice cultivation to improve soil productivity. However, little is known about the role of microbes under legume rotation in affecting soil productivity. To elucidate this, a long-term paddy cropping experiment was set up to study the relationship between crop yield, soil chemical properties, and key microbial taxa under a double-rice and milk vetch rotation. Milk vetch rotation significantly improved soil chemical properties compared to no fertilization treatment, and soil phosphorus was a major factor correlated with crop yield. Long-term legume rotation increased soil bacterial alpha diversity and changed soil bacterial community. After milk vetch rotation, the relative abundances of *Bacteroidota*, *Desulfobacterota*, *Firmicutes*, and *Proteobacteria* increased while those of *Acidobacteriota*, *Chloroflexi*, and *Planctomycetota* decreased. Moreover, milk vetch rotation increased the relative abundance of phosphorus-related gene K01083 (*bpp*), which was significantly correlated with soil phosphorus content and crop yield. Network analysis showed that taxa of *Vicinamibacterales* were positively correlated with total phosphorus and available phosphorus, which was a potential taxon contributing to the availability of soil phosphorus stock. Our results indicated that milk vetch rotation could enrich key taxa with latent phosphate-solubilizing ability, increase the content of soil available phosphorus, and finally enhance crop yield. This could provide scientific guidance for better crop production.

## 1. Background

Rice is a crucial staple crop for more than half of the world’s population, and China is a significant producer, responsible for 18.5% of global cultivation area and 28.0% of global production output. Ensuring sustainable rice production is imperative for global food security (Zhou et al., 2020). Increasing demand to feed the huge population is driving the search for more efficient and eco-friendlier cropping regimes (Godfray et al., 2010), and legume rotation is a widely applied measure (Zhao et al., 2022). With a long planting history, legume manure has an integrative effect on soil abiotic properties and crop production (Yang et al., 2022). The symbiotic legume root nodules with rhizobium are responsible for soil nitrogen fixation, improving soil nitrogen supply (Dong et al., 2021). Additionally, legumes can take up nutrients in deeper soil due to deep roots and supply these nutrients after being plowed into soil (Xue et al., 2016). Milk vetch is an important leguminous crop in traditional agricultural production. It can improve the effectiveness of soil nutrients and promote the absorption and accumulation of nutrients by crops (Solangi et al., 2019). However, there is limited knowledge about the biotic effect of legume rotations. Since soil microbes play crucial roles in soil nutrient cycling (Jiao et al., 2019), determining how soil microbes and their functions respond to legume rotation can help to accurately predict changes in soil productivity.

Crop rotation can regulate the soil microbial community, mainly through specific root exudates and litter input (Panettieri et al., 2020), and consequently enrich specific microbial consortia (Xiong and Lu, 2022) and perform different functions. For example, the selective effect of specific substrates on microbial communities can induce a shift of nutrient metabolism *via* changing the activity of certain enzymes (Peng et al., 2023). Research on community-level sole-carbon-source utilization properties of soil microbes showed that legume rotation altered the carbon source preference of the microbial community (Aschi et al., 2017). However, determining the general function of microbes could mask the heterogeneous roles of key microbes in soil nutrient cycling under crop rotation. The prediction of microbial community genes based on the phylogenetic similarity between taxa and reference sequences could be used to produce a latent abundance table containing information on microbial multiple functions (Czech et al., 2022), thus shedding light on the possible shift in microbial metabolism after legume rotation.

To investigate the selective effect of legume rotation on microbial consortia and their reaction to soil nutrient cycling and crop production, we performed a long-term legume rotation experiment. The relationships between soil key microbial taxa, chemical properties, and rice yield under different cropping regimes were determined. The phylogenetic prediction method for bacterial function genes was also applied to determine the reaction of key taxa to nutrient cycling. We hypothesized that long-term legume rotation would enrich specific microbial groups with specific functions. We further hypothesized that the enriched functional groups have potential to alter soil nutrient cycling after legume rotation.

## 2. Materials and methods

### 2.1. Site description and sampling

The samples were collected from a long-term paddy cropping experiment site in Yingtan City (28°15′30″N, 116°55′30″E), Jiangxi Province in China. This site has a subtropical monsoon climate with mean temperature of 17.6°C and mean precipitation of 1795 mm annually. The soil of the site is paddy soil developed on quaternary red clay. Double-cropped rice was cultivated on the site for about 30 years, and all agronomic practices were very similar between treatments except for cropping regime.

Three cropping and fertilization regimes with three replications were applied: (1) CK, double-rice with no fertilization; (2) NPK, double-rice with mineral fertilization (230 kg N, 136 kg P_2_O_5_, and 84 kg K_2_O per ha per year); and (3) NPKGM, double-rice and milk vetch rotation with mineral fertilization (230 kg N, 136 kg P_2_O_5_, and 84 kg K_2_O per ha per year). Milk vetch (*Astragalus sinicus* L.) was planted after harvest of the later rice crop and plowed during flowering stage, where 5,000 kg of fresh milk vetch (dry weight 2,500 kg) ha^−1^ year^−1^ was applied.

The later rice crop was harvested, husked, and weighed in November 2017, and the rice yield of each plot was calculated after measuring the water content. Five surface soil samples (0–20 cm) were collected using an earth-boring auger in a ‘X’ pattern and merged into one soil sample for each plot after rice harvest. Each soil sample was sieved through 2 mm and divided into two parts: one part was air-dried for physiochemical assay, and the other was stored at −40°C for DNA extraction.

### 2.2. Soil chemical property measuring

Soil pH was determined using a pH meter in a 1:2.5 of soil and water suspension. The total nitrogen (TN) content in soil was determined using the Kjeldahl method (Pansu and Gautheyrou, 2006). The available nitrogen (AN) was determined using the alkali hydrolysis and micro diffusion method (Pansu and Gautheyrou, 2006). Total phosphorus (TP) and available phosphorus (AP) were determined by vanadium-molybdate photometric method, and total potassium (TK) and available potassium (AK) were determined by inductively coupled plasma-atomic emission spectrometry (Pansu and Gautheyrou, 2006). Soil organic carbon (SOC) was determined using potassium dichromate oxidation with an external heating method (Pansu and Gautheyrou, 2006).

### 2.3. Soil DNA extraction and sequencing

Each 1 g soil sample was weighed, and soil microbial DNA was extracted according to the manufacturer’s instructions using FastDNA™ Spin Kit (MP Biomedicals, Santa Ana, CA, USA). The hyper-variable region (V4–V5) of prokaryotic 16S rRNA was amplified using the primers 515F (5′-GTGCCAGCMGCCGC GGTAA-3′) and 907R (5′-CCGTCAATTCCTTTGAGTTT-3′) with barcodes. All PCR reactions were carried out with 15 μL of Phusion^®^ High-Fidelity PCR Master Mix (New England Biolabs), 0.2 μM of forward and reverse primers, and about 10 ng of template DNA. Thermal cycling consisted of initial denaturation at 98°C for 1 min, followed by 30 cycles of denaturation at 98°C for 10 s, annealing at 50°C for 30 s, and elongation at 72°C for 30 s, with a final step of 72°C for 5 min. The samples after PCR amplification were sequenced on an Illumina NovaSeq platform, and 250-bp paired-end reads were generated. The raw reads were denoised, dereplicated, and clustered using a VSEARCH pipeline. High-quality sequences of 84,811–141,764 reads per sample were obtained, and we finally obtained 12,913 operational taxonomic units (OTUs). A trained Naïve Bayes classifier based on the Silva 138 database was deployed to classify the OTU sequences (Quast et al., 2013; Bolyen et al., 2019). After filtering the non-bacterial sequences, 12,673 OTUs were prepared for downstream analysis. All the raw sequences tested in the study were uploaded to the NCBI SRA database (accession no., PRJNA957222).

Bacterial community gene abundances were predicted using Phylogenetic Investigation of Communities by Reconstruction of Unobserved States (Douglas et al., 2020), and a final list of 7,425 latent function genes and their abundances were obtained. The shifts of three phosphorus-related genes (K00117, *gcd*; K01083, *bpp*; and K06137, *pqqC*) and taxa contributing up to 80% of their abundance were analyzed.

### 2.4. Data analysis

One-way analysis of variance was applied to the differences between soil chemical properties and crop yields among treatments. Pearson correlations were calculated using function ‘*rcorr*’ of the R package ‘*Hmisc.*’ The bacterial Shannon and richness indices were calculated using functions ‘*diversity*’ and ‘*estimateR*’ of the R package ‘*vegan*,’ respectively. Constrained principal coordinate analysis (CPCoA) was adopted to display the discrepancies of bacterial community structure between treatments using function ‘*capscale*’ of the R package ‘*vegan.*’ A network containing TP, AP, rice yield, and OTUs contributing to the previously selected phosphorus-related genes was constructed based on the Pearson correlations with *p* < 0.001 (Supplementary Figure S1). Subnetworks characterizing correlations per sample were obtained later, and topological indexes of every subnetwork were calculated (Supplementary Table S1). To determine the functional groups related to nutrient cycling, a subnetwork containing TP, AP, rice yield, and taxa directly related to them was obtained from the initial network. The network was constructed using the R package ‘*igraph*,’ and the network graphic was constructed using the Gephi program.

## 3. Results

### 3.1. Effect of milk vetch rotation on soil chemical properties and rice yield

The NPK and NPKGM treatments increased soil nutrient content and rice yield. The NPKGM significantly increased pH, SOC, TN, TP, AP, AK, and rice yield compared to CK (*p* < 0.05) (Table 1).

**Table 1:** Soil physiochemical properties and rice yield after different fertilization treatments.

Treat	pH	SOC	TN	TP	TK	AN	AP	AK	Yield
	1:2.5	g kg^−1^	g kg^−1^	g kg^−1^	g kg^−1^	mg kg^−1^	mg kg^−1^	mg kg^−1^	kg/ha
CK	5.59 ± 0.03b	7.69 ± 0.88b	0.93 ± 0.07b	0.86 ± 0.07b	15.21 ± 0.71a	77.18 ± 7.35a	44.39 ± 6.13b	92.50 ± 21.36b	4391.08 ± 478.15b
NPK	6.42 ± 0.10a	8.75 ± 1.64ab	1.09 ± 0.26ab	1.41 ± 0.15ab	14.07 ± 1.46a	95.55 ± 24.10a	88.56 ± 9.22a	202.50 ± 19.53a	6692.23 ± 340.47a
NPKGM	6.60 ± 0.10a	10.35 ± 1.26a	1.32 ± 0.11a	1.56 ± 0.04a	14.99 ± 1.19a	101.68 ± 9.25a	99.40 ± 8.07a	237.50 ± 43.37a	7059.08 ± 393.03a

SOC, soil organic carbon; TN, total nitrogen; TP, total phosphorus; TK, total potassium; AN, available nitrogen; AP, available phosphorus; AK, available potassium; CK, double-rice with no fertilization; NPK, double-rice with mineral fertilization (230 kg N, 136 kg P_2_O_5_, and 84 kg K_2_O per ha per year); and NPKGM, double-rice and milk vetch rotation with mineral fertilization (230 kg N, 136 kg P_2_O_5_, and 84 kg K_2_O per ha per year). Different letters indicate a significant difference between treatments at *p* < 0.05.

**Figure 1:**
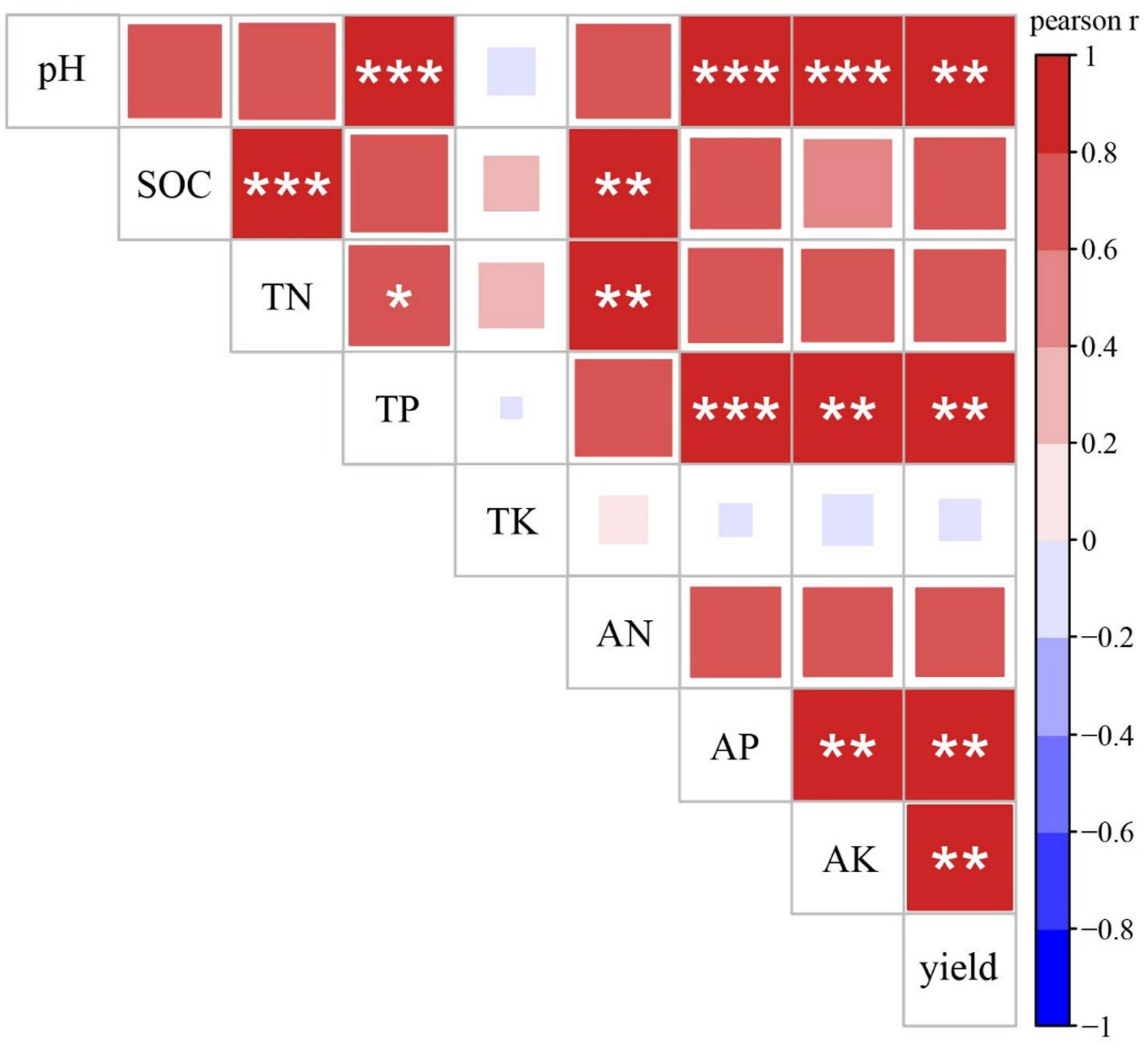
Pearson correlations between crop yield and soil properties. SOC, soil organic carbon; TN, total nitrogen; TP, total phosphorus; TK, total potassium; AN, available nitrogen; AP, available phosphorus; AK, available potassium.

Pearson correlation analysis showed associations between rice yield and soil chemical properties (Figure 1). Among all soil chemical properties, pH, TP, AP, and AK were significantly correlated with crop yield, and pH was significantly correlated with TP, AP, and AK.

### 3.2. Effect of milk vetch rotation on soil bacterial community structure

**Figure 2:**
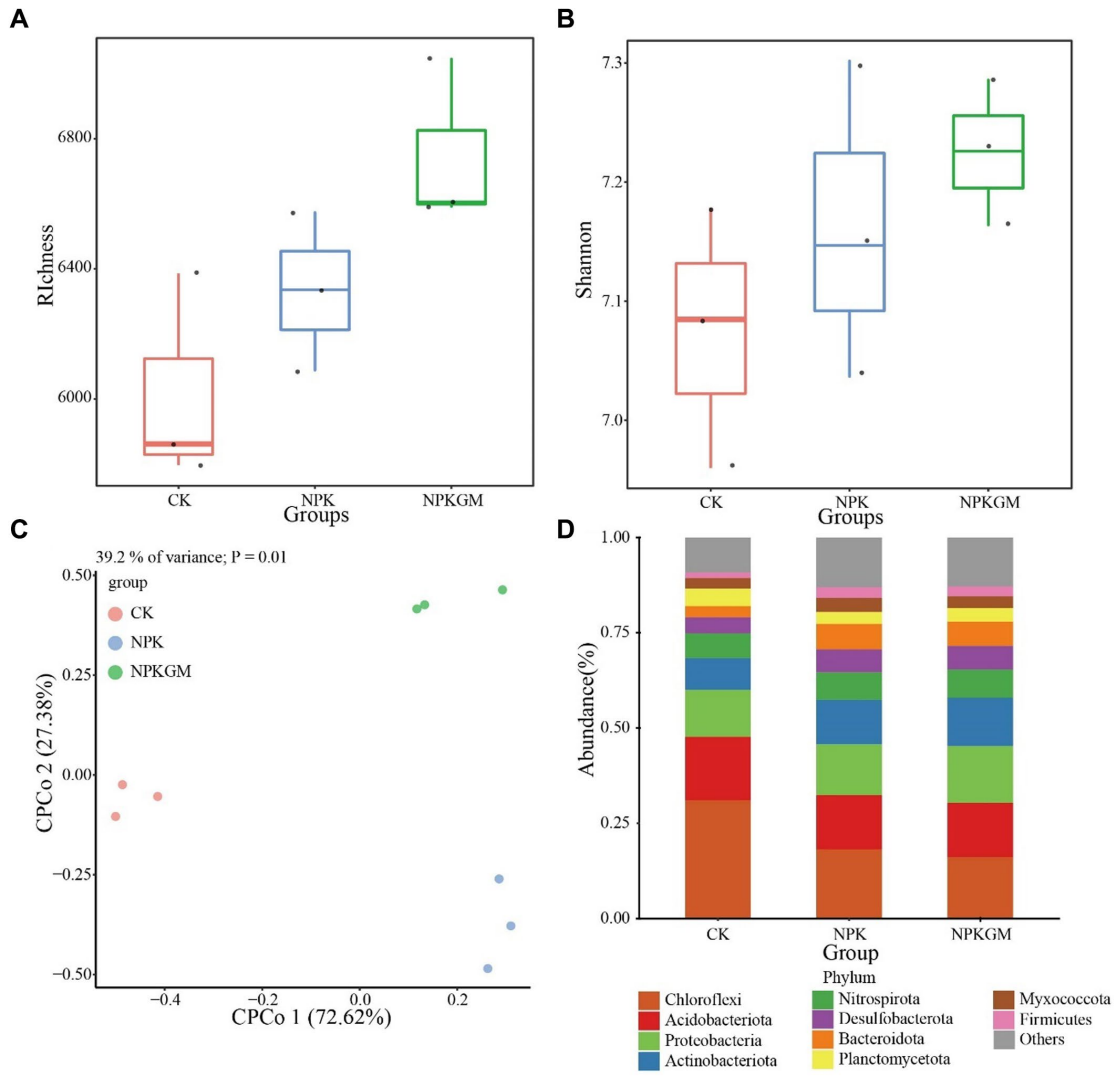
Bacterial community **(A,B)** diversity and **(C,D)** structure under different fertilization treatments: **(A)** richness index, **(B)** Shannon index, **(C)** CPCoA of soil bacterial community under different fertilization treatments, and **(D)** soil bacterial composition in phylum level under different fertilization treatments. CK, double-rice with no fertilization; NPK, double-rice with mineral fertilization (230 kg N, 136 kg P_2_O_5_, and 84 kg K_2_O per ha per year); and NPKGM, double-rice and milk vetch rotation with mineral fertilization (230 kg N, 136 kg P_2_O_5_, and 84 kg K_2_O per ha per year).

Although not significant, the richness and Shannon indices of the bacterial community were higher in the NPKGM than in CK and NPK treatments (Figures 2A,B). The CPCoA showed that the bacterial community changed in the different cropping regimes (Figure 2C), with principal coordinates 1 and 2 explaining 76.62 and 27.38% of the total variance, respectively. Further analysis showed that the legume rotation increased the relative abundances of *Bacteroidota*, *Desulfobacterota*, *Firmicutes*, and *Proteobacteria* but reduced those of *Acidobacteriota*, *Chloroflexi*, and *Planctomycetota* (Figure 2D).

### 3.3. Effect of milk vetch rotation on phosphorus-related genes

**Figure 3:**
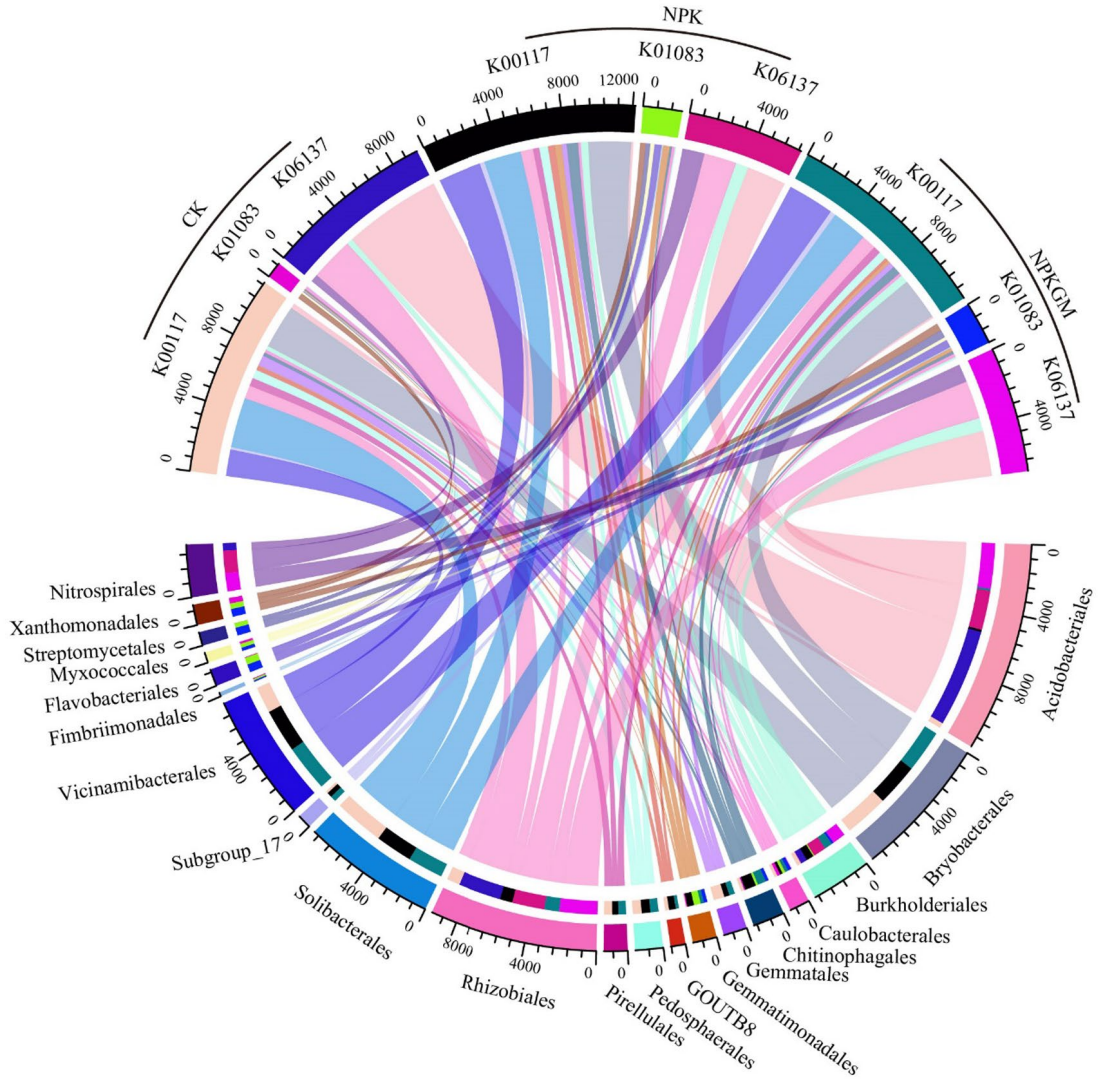
Circular plot of abundance of phosphorus-related genes K00117 (*gcd*), K01083 (*bpp*), and K06137 (*pqqC*) and the average abundance of genes contained by taxa. CK, double-rice with no fertilization; NPK, double-rice with mineral fertilization (230 kg N, 136 kg P_2_O_5_, and 84 kg K_2_O per ha per year); and NPKGM, double-rice and milk vetch rotation with mineral fertilization (230 kg N, 136 kg P_2_O_5_, and 84 kg K_2_O per ha per year).

The predicted absolute abundances of the enriched genes were in the following order in all treatments: K00117 (*gcd*) > K06137 (*pqqC*) > K01083 (*bpp*) (Figure 3). The abundance of K00117 was highest in NPK and lowest in CK. The abundance of K06137 was highest in CK and lowest in NPK. Moreover, abundance of K01083 was higher in NPKGM than in CK and NPK. Correlation analysis showed that K00177 had no significant correlation with TP, AP, or crop yield (*p* > 0.05, Supplementary Figure S1); however, K01083 was significantly positively correlated (and K06137 was negatively correlated) with TP, AP, and crop yield (*p* < 0.05).

*Bryobacterales*, *Solibacterales*, *Vicinamibacterales*, *Rhizobiales*, and *Pedosphaerales* were the top five taxa contributing most to K00117 abundance. *Xanthomonadales*, *Flavobacterales*, *Streptomycetales*, *Gemmatimonadales*, and *Myxococcales* were the top five taxa contributing most to K01083. *Acidobacteriales*, *Rhizobiales*, *Nitrospirales*, *Burkholderiales*, and *Caulobacterales* were the top five taxa contributing most to K06137. *Bryobacterales*, *Solibacterales*, and *Pedosphaerales* contributed more K00117 genes in CK, while *Vicinamibacterales* and *Rhizobiales* contributed more K00117 in NPKGM. *Gemmatimonadales* and *Myxococcales* contributed more K01083 genes in NPK, while *Xanthomonadales*, *Flavobacterales*, and *Streptomycetales* contributed more K01083 in NPKGM. *Acidobacteriales* and *Rhizobiales* contributed more K06137 genes in CK, while *Nitrospirales* contributed more K06137 genes in NPK. *Burkholderiales* and *Caulobacterales* contributed more K06137 in NPKGM.

### 3.4. Relationships between soil phosphorus content, crop yield, and phosphorus-related taxa

**Figure 4:**
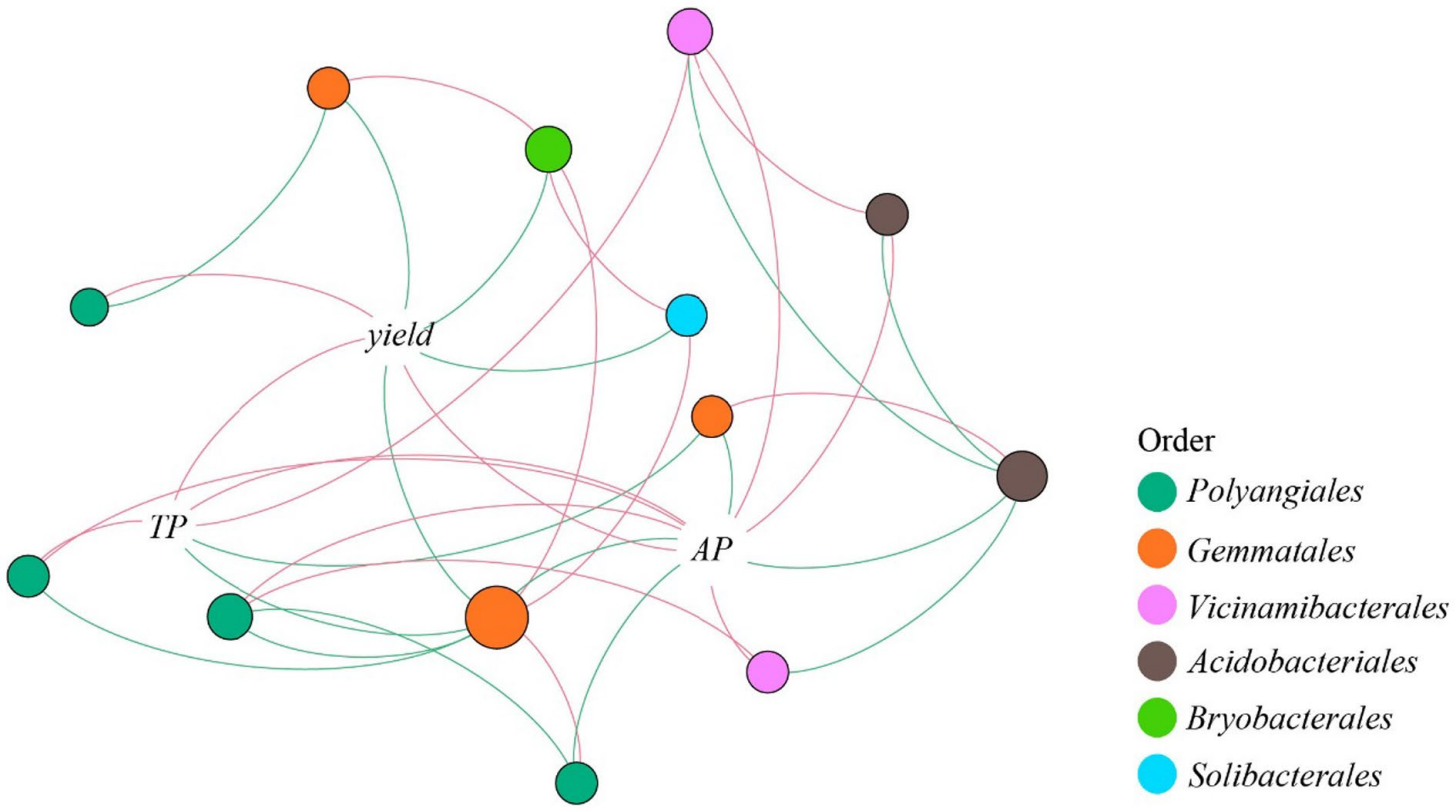
Network analysis of correlativity between TP, AP, yield, and phosphorus gene related taxa. Red edges indicate positive correlation, while green edges indicate negative correlation. The size of a node indicates the number of edges it participates in. TP, total phosphorus; AP, available phosphorus.

The Balaban Index and Topological Index showed no significant difference between treatments, while the positive edges/negative edges ratio in CK was significantly higher than other treatments (Supplementary Table S1). Thirteen OTUs belonging to six orders were directly correlated with TP, AP, or crop yield, and four orders were correlated with TP, AP, or both (Figure 4). Nodes belonging to *Gemmatales* were negatively correlated with TP, AP, and crop yield, while nodes belonging to *Vicinamibacterales* were positively correlated with TP and AP. From the order *Polyangiales*, OTU 6233 had a positive correlation with TP and AP, while OTU 8505 was negatively correlated with AP. The OTU 2853 was positively correlated with AP, while another *Acidobacteriales* (OTU 7944) was negatively correlated with AP (Supplementary Figure S2).

## 4. Discussion

### 4.1. Rotation of milk vetch changed soil chemical properties and crop yield

Consistent with previous research (Zhang et al., 2023), long-term rotation of milk vetch increased double-rice yield. The significant positive relationship between rice yield and phosphorus content indicates that the higher phosphorus level was responsible for the high productivity of the rotation soil. The research site is subtropical China, which is dominated by phosphorus limitation (Yu et al., 2018), thus, AP supply could enhance crop production. As well as phosphorus content, pH and AK were positively related to crop yield. Soil pH is a crucial factor regulating soil nutrient availability (Xue et al., 2019) and the microbial community (Liu et al., 2018), while the potassium stock in subtropical red soil experiences strong leaching, leading to demand for potassium. Therefore, improving soil pH and K availability likely led to the yield increase.

Milk vetch rotation increased soil phosphorus content, similar to results of Li et al. (2018). Researchers found that legume rotation improved the humification of SOC (Virk et al., 2021) and could inhibit phosphorus migration, activate fixed phosphorus, improve the distance of phosphorus movement, and finally improve the effective phosphorus content in soil (Xu et al., 2017; Wulanningtyas et al., 2021). The higher pH in the rotation treatment also likely improved phosphate availability (Barrow and Lambers, 2022). Moreover, the milk vetch roots could reach deeper soil, providing more phosphorus to the surface soil (Zhang et al., 2022).

### 4.2. Rotation of milk vetch changed soil bacterial community

Cropping regimes such as legume rotation have important effects on bacterial community diversity and composition (Zheng et al., 2021). Similar phenomena were found in our study, and the higher richness and Shannon indices in the NPKGM treatment indicated that the milk vetch rotation increased microbial diversity (Benkwitt et al., 2020). We found that milk vetch rotation increased the relative abundance of high nitrogen affinity taxon *Bacteroidota*, possibly due to the higher TN content in the NPKGM treatment (Li W. L. et al., 2022), and *Proteobacteria*, which is the source of many enzymes and usually participates in the biological cycling of mineral elements in soil to maintain soil fertility (Chaudhry et al., 2012; Li W. L. et al., 2022). In addition, both *Bacteroidota* and *Proteobacteria* are *eutrophic* bacteria, which benefit from eutrophic environments in the NPKGM treatment (Fierer et al., 2007). Related studies show that lower soil pH led to higher *Firmicutes* abundance (Li Y. Z. et al., 2022; Palansooriya et al., 2022), which contrasts with our study in which *Firmicutes* was enriched in NPKGM at higher pH. *Firmicutes* can also benefit from a high source-availability environment, whose content increases with abundance of nutrients (Vanwonterghem et al., 2016; Zhou et al., 2021); in our study, *Firmicutes* may have responded more to nutrients than pH.

Hu et al. (2022) found that *Chloroflexi* was negatively correlated with TN content, consistent with our results of lower *Chloroflexi* abundance in the NPK and NPKGM treatments. As typical *copiotrophic* taxa, both *Acidobacteria* and *Planctomycetota* usually dominate in barren conditions (Fierer et al., 2007; Shelyakin et al., 2022) and thus were enriched in our CK treatment.

### 4.3. Keystone taxa and their predicted function promoted soil phosphorus metabolism under double-rice and milk vetch rotation

Unlike the recognition that phosphate-solubilizing microorganisms were responsible for soil AP (Chen et al., 2023), K00117 (*gcd*) and K06137 (*pqqC*), known as genes that encode enzymes to mediate the resolving of soil phosphate, had non-significant or even negative correlations with soil AP and TP in CK or NPK in our study. Only K01083 (*bpp*) was positively correlated with AP and TP in NPKGM. A possible mechanism was the phosphate released from soil minerals being easily leached from red paddy soil under high precipitation (Peng et al., 2020), while the organic phosphorus released from milk vetch rotation could remain longer in soil (Zhao et al., 2013), hence we observed a significant positive correlation between K01083 and soil phosphorus content even when the abundance of K01083 was low (Jorquera et al., 2013). In addition, encoding phytases that catalyze the hydrolysis of plant-sourced phytate (Lu et al., 2022) and taxa containing *bpp* could benefit in soil with greater plant litter input (Liu X. et al., 2022). This can also further explain the enrichment of *bpp* in the NPKGM treatment.

Moreover, evidence showed that high pH and high AP content could reduce the abundances of taxa with genes encoding *phoD* such as *Gemmatales*, since we found a negative correlation between AP and *Gemmatales*. Nodes belonging to *Acidobacteriales* had an opposite correlation with AP and AP-related taxa, indicating that different specific taxa belonging to *Acidobacteriales* might play different roles in phosphorus metabolism (Cui et al., 2022; Liu H. et al., 2022). Strong correlations were found between *Polyangiales* and AP (Figure 3). As a predator belonging to the phylum *Myxococcota*, it can be inferred that *Polyangiales* might benefit in a high-nutrition environment which provides more microorganisms. Among the taxa concerned with phosphorus content, *Vicinamibacterales* was the only taxon that was purely positively correlated with AP and rice yield in the network analysis. It was reported that *Vicinamibacterales* could encode *pit* that participated in the assimilation of phosphate and prevented the loss of AP in an extreme leaching environment (Wu et al., 2022). Our study indicated that legume rotation may influence soil element cycling, especially phosphorus cycling, by enriching certain key species and key functional genes to increase phosphorus availability and ultimately promote crop yield.

## 5. Conclusion

Our milk vetch rotation improved the physiochemical properties and crop yield of the paddy soil. In addition, this rotation altered the soil bacterial community, especially some groups closely associated with phosphorus-related functional genes. Network analysis showed that taxa belonging to six phyla were associated with TP, AP, and crop yield. Taken together, milk vetch rotation could enrich key taxa encoding phytase, increase soil AP content, and ultimately improve crop yield. This could provide scientific guidance for better crop production. However, we were mainly interested in the relationship between changes and interactions of soil characteristics and microbial communities and crop yield. In future studies, it is recommended to focus on the expression of relevant functional genes to obtain direct evidence that nutrient cycling is closely related to rice yield.

## Data availability statement

The original contributions presented in the study are included in the article/Supplementary materials, further inquiries can be directed to the corresponding author.

## Author contributions

MX: measurement and analysis, writing–original draft, and visualization. XM: methodology, data curation, and formal analysis. JL: methodology, review, and editing. MW: software and formal analysis. ZL: funding acquisition and validation. ML: funding acquisition, review, and editing. All authors contributed to the article and approved the submitted version.

## Funding

This work was supported by the National Natural Science Foundation of China (42177294), the Key Research and Development Program of Jiangxi Province (20212BBF63007), the Central Guidance for Local Science and Technology Development Projects (20231ZDD02003), China Agriculture Research Systems of MOF and MARA (CARS-22), and the Jiangxi Modern Agricultural Innovation Project (JXXTCXQN202008).

## Conflict of interest

The authors declare that the research was conducted in the absence of any commercial or financial relationships that could be construed as a potential conflict of interest.

The reviewer YJ declared a shared affiliation with the authors MX, XM, ML, MW, and ZL to the handling editor at the time of review.

## Publisher’s note

All claims expressed in this article are solely those of the authors and do not necessarily represent those of their affiliated organizations, or those of the publisher, the editors and the reviewers. Any product that may be evaluated in this article, or claim that may be made by its manufacturer, is not guaranteed or endorsed by the publisher.
