# Supplementary material for: Potential effect of key soil bacterial taxa on the increase of rice yield under milk vetch rotation
Source: Front Microbiol. 2023 May 22;14:1150505. doi: 10.3389/fmicb.2023.1150505 (PMC10241072; doi:10.3389/fmicb.2023.1150505)
Supplement: Supplementary file 1 [file Data_Sheet_1.docx]

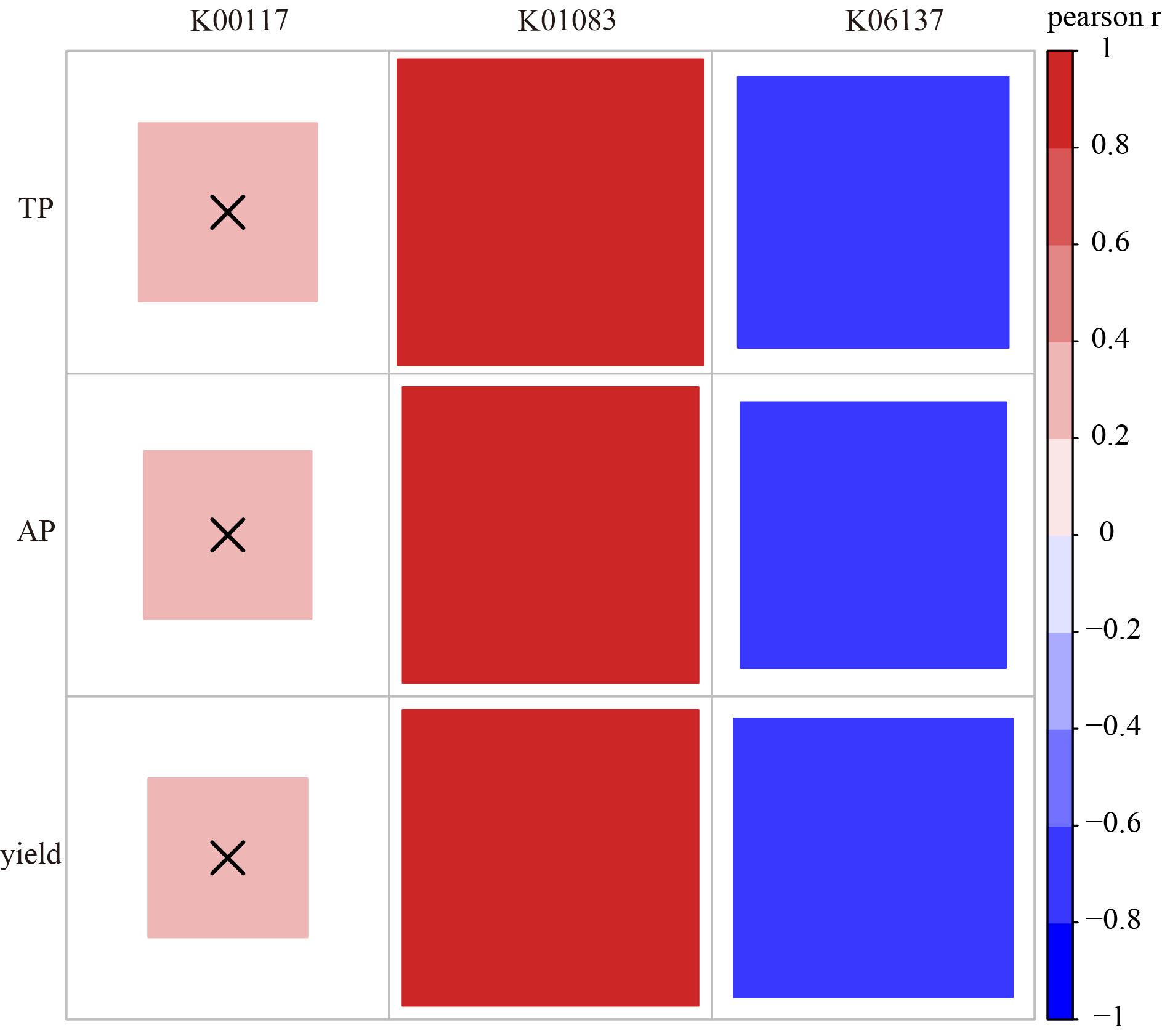


Fig. S1 Pearson correlations between phosphorus related genes (K00117: *gcd*, K01083: *bpp*, K06137: *pqqC*) and TP, AP, and crop yield. TP, total phosphorus; AP, available phosphorus.

Table S1 Topological index of initial network and subnetworks in each treatment

| **Treat** | **Nodes number** | **Edges number** | **Positive edges/Negative edges** | **Average degree** | **Average path length** | **Betweenness centralization** |
| --- | --- | --- | --- | --- | --- | --- |
| Initial net | 1775 | 12260 | 1.73 | 13.81 | 5.05 | 0.02 |
| CK | 1215.67±13.20a | 7569.33±531.58a | 2.27±0.29a | 12.45±0.78a | 5.1±0.17a | 0.03±0.00a |
| NPK | 1243±77.62a | 6746.67±722.08a | 1.54±0.11b | 10.84±0.73a | 5.09±0.08a | 0.02±0.01a |
| NPKGM | 1300.67±68.70a | 7844.67±1276.12a | 1.65±0.23b | 12.03±1.47a | 5.19±0.10a | 0.02±0.00a |

Different letters indicate significant difference between treatments at the 0.05 level. CK, double-rice with no fertilization; NPK, double-rice with mineral fertilization (230 kg N, 136 kg P_2_O_5_, and 84 kg K_2_O per ha per year); and NPKGM, double-rice and milk vetch rotation with mineral fertilization (230 kg N, 136 kg P_2_O_5_, and 84 kg K_2_O per ha per year).


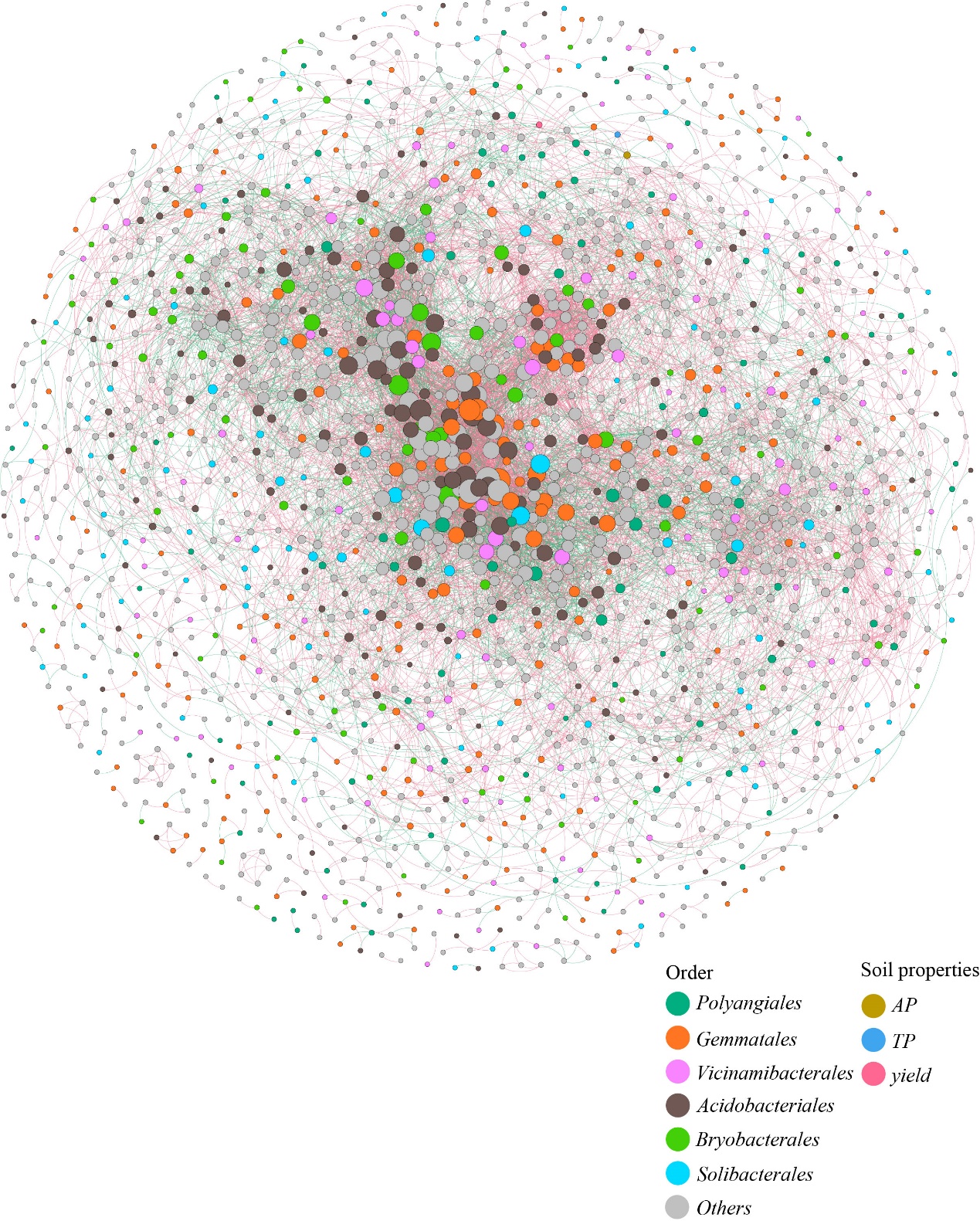


Fig. S2 Network based on the pearson correlation between TP, AP, rice yield, and OTUs contributed to phosphorus related genes (K00117: *gcd*, K01083: *bpp*, and K06137: *pqqC*), the colors of the OTUs indicated the orders they belonged to. TP, total phosphorus; AP, available phosphorus.
